# Effect of bioactive glass toothpaste on the white spots around orthodontic brackets: A systematic review and meta-analysis

**DOI:** 10.34172/joddd.025.41686

**Published:** 2025-03-31

**Authors:** Arezoo Jahanbin, Reyhaneh Hanaei, Erfan Bardideh, Maryam Omidkhoda, Farnaz Zia

**Affiliations:** ^1^Department of Orthodontics, School of Dentistry, Mashhad University of Medical Sciences, Mashhad, Iran; ^2^General Dental Practitioner, School of Dentistry, Mashhad University of Medical Sciences, Mashhad, Iran; ^3^Orthodontist, Dental Research Center, School of Dentistry, Mashhad University of Medical Sciences, Mashhad, Iran

**Keywords:** Bioactive glass toothpaste, Orthodontics, White spot lesions

## Abstract

**Background.:**

White spot lesions (WSLs), early indicators of tooth decay, are common in patients with fixed orthodontic appliances and can disrupt esthetic outcomes. Various methods have been proposed to prevent and treat WSLs, with bioactive glass products being one of the latest approaches. This study aims to evaluate the effect of bioactive glass toothpastes on WSLs around orthodontic brackets.

**Methods.:**

Relevant articles were identified using databases such as PubMed, EMBASE, Cochrane’s CENTRAL, Scopus, and Web of Science up to November 2023. The full texts of selected studies were retrieved, and their quality was assessed. The study included clinical and in vitro research. Four clinical studies (2015‒2023) were reviewed, with meta-analysis performed on three. A random-effects inverse variance meta-analysis was performed, and the quality of the evidence was graded using GRADE.

**Results.:**

No significant difference was found between bioactive glass and fluoride toothpaste in remineralizing WSLs (*P*=0.10, SMD=-0.29). Nine in vitro studies (2013‒2022) were reviewed, with a meta-analysis on three showing significant lesion depth reduction with bioactive glass compared to no treatment (*P*<0.00001, MD=-63.98).

**Conclusion.:**

Bioactive glass toothpaste may be effective in remineralizing WSLs, though its efficacy is not significantly different from conventional fluoride toothpaste.

## Introduction

 Dental caries is one of the most common chronic diseases worldwide. White spots indicate the initial stages of caries, beginning on tooth enamel.^1^ The first signs of enamel loss are demineralized areas that appear as white spots near the gingival margin. If this process continues, it can lead to cavities. Enamel decalcification/demineralization is undoubtedly one of the most significant challenges during orthodontic treatment. Since one of the main goals of orthodontic treatment is esthetics, these lesions can affect the final outcome of the treatment. The best approach during orthodontic treatment is to prevent white spots before they occur. Dentists use various techniques to prevent and treat white spots, including encouraging oral hygiene, using topical fluoride, casein phosphopeptide-amorphous calcium phosphate (CPP-ACP), antimicrobial products, tooth bleaching, microabrasion, and resin infiltration.^2^

 One of the materials used today to treat white spots is bioactive glasses (BG). These highly biocompatible materials are silicate-based and can form a strong chemical bond with tissues.^3^ Bioactive glasses are derived from the family of calcium phosphosilicates, which can be decomposed in body fluids such as blood and saliva. These materials are useful for repair and remineralization.^4^

 Bioactive glasses have broad uses, especially in dentistry, such as treating tooth sensitivity or maintaining bone after tooth extraction. One common use of bioactive glasses in dentistry is enamel remineralization. Primary carious lesions, like white spots, can be remineralized and restored through regular tooth cleaning, plaque removal, and fluoride application. Studies have shown that BG may be more effective than fluoride and CPP-ACP in white spot remineralization.^5^ The novelty of the current paper is to investigate the effects of bioactive glass-containing toothpaste on WLS around orthodontic brackets through a systematic review.

## Methods

###  Study design 

 PICO in this study is defined as follows:

 P: Patients or teeth that have undergone fixed orthodontic treatment

 I: Using toothpastes containing bioactive glass

 C: Using toothpastes without bioactive glass

 O: Investigating the remineralization ability of toothpastes containing bioactive glass on white spot lesions (WSLs) around brackets

###  Search strategy and sources

 Due to the lack of qualified clinical studies, a review was also conducted on in vitro studies. Therefore, this article includes two parts: the first part for clinical studies and the second part for in vitro studies (Table 1).

**Table 1:** Databases applied search strategy, and numbers of retrieved studies

**Database of published trials, dissertations and conference proceedings**	**Search strategy used**	**Hits**
MEDLINE searched via PubMed searched on October 27, 2023^th^, via https://www.ncbi.nlm.nih.gov/	#1 bioactive glass OR bioglass OR novamin OR 45S5 OR bioactive paste OR bioactive #2 orthodontic OR orthodontics OR brackets #3 #1 AND #2	449
Web of Science Core Collection was searched via Web of Knowledge on November 1, 2023^th^, via apps.webofknowledge.com	#1 TS = (bioactive glass OR bioglass OR novamin OR 45S5 OR bioactive paste OR bioactive) #2 TS = (Orthodontics OR bracket) #3 #1 AND #2	72
EMBASE searched via Ovid on November 1, 2023^th^, via https://www.embase.com/	#1 ('novamin'/exp OR 'novamin' OR 45s5 OR 'bioactive glass'/exp OR 'bioactive glass' OR 'bioglass'/exp OR 'bioglass' OR 'bioglass 45s5'/exp OR 'bioglass 45s5' #2 'orthodontics'/exp OR orthodontics OR brackets #3 #1 AND #2	96
Scopus searched via Scopus on October 28, 2023^th^, via https://www.scopus.com	TITLE-ABS-KEY (bioactive AND glass OR bioglass OR novamin OR 45s5 OR bioactive AND paste OR bioactive) AND TITLE-ABS-KEY (orthodontics OR brackets)	84
Cochrane Central Register of Controlled Trials searched via the Cochrane Library Searched on October 31, 2023^th^, via https://www.cochranelibrary.com/	#1 bioactive glass 249 #2 bioglass 39 #3 bioactive paste 34 #4 orthodontic 5088 #5 (#1 OR #2 OR #3) AND #4 17	17
Total		718

###  The first part (clinical)

*Inclusion criteria:* (1) Articles studying the remineralization properties of toothpastes containing bioactive glass; (2) The target group of the study comprising a human sample.

*Exclusion criteria:* (1) Articles that did not have a control group; (2) Articles that assessed other anti-caries and remineralizing materials except for bioactive glasses; (3) Articles that evaluated patients without orthodontic treatment; (4) Articles involving animals.

###  The second part (in vitro)

*Inclusion criteria:* (1) Articles studying the remineralization properties of bioactive glasses; (2) The target group of the study involving healthy, intact extracted teeth.

*Exclusion criteria:* (1) Articles that did not have a control group; (2) Articles that reviewed other anti-caries and remineralizing materials except for bioactive glasses; (3) Articles whose structure did not contain bioactive glass, toothpaste, or paste.

###  Study selection and data extraction

 In the first step, a systematic search strategy was designed using keywords related to the study topic. Then, a comprehensive search was run in PubMed, Scopus, Embase, Web of Science, and Cochrane Central Register of Controlled Trials databases, and all the obtained articles were reviewed. The titles and abstracts of related studies were examined by two researchers (RH & EB) separately, and according to the inclusion and exclusion criteria, the studies were excluded. Any disagreements between these two researchers were resolved by the third researcher (AJ). The full texts of the remaining studies were obtained and analyzed to enter the review and meta-analysis process. The data extraction of the selected articles was performed by one researcher (RH), and its correctness was checked by another researcher (EB). The desired information was extracted from the included studies, including the name of the authors of the study, the location of the study, the year of publication of the articles, the number of patients in the treatment and control groups, the average age of the patients, the gender of the participants in the studies, the duration of the study, the inclusion and exclusion criteria of the study, the type of treatment for WSLs, the method of measuring the intensity of lesions and the results of treatment, the fluorescence of enamel with white spots before and after treatment, the ratio of calcium and phosphorus to each other, the depth of WSLs, and the difference in the chemical structures of the depth of WSLs (Tables 2 and 3).

**Table 2:** A summary of the characteristics of the included in vivo studies

**First Author/ date**	**Study design**	**Duration**	**Age**	**Novamin added to**	**Time points**	**Sample size**		**Gender**		**Groups**	
Hoffman, 2015^6^	Clinical trial	6 months	12-25 years	Toothpaste	T1: 3 months	T0	48	flo	15M/9F	Control	Fluoride
					T2: 6 months	T1	44	Nov	17M/7F	Experiment	Novamin
Mollabashi, 2022^7^	Clinical trial	6 months	15-30 years	Fluoride toothpaste	T1: 1 month	T0	38			Control	Fluoride
					T2: 3 months	T1,2	36			Test	Fluoride and Novamin
Salah, 2022^8^	Clinical trial	6 months	14-26 years	Toothpaste	T1: 1 week	T0	60	T0	39F 21M	BioMinF (Bio-BAG)	
					T2: 1 month					NovaMin (N-BAG)	
					T3: 3 months	T4	56	T4	37F 19M		
					T4: 6 months					CPP-ACP (control group)	
Tiwari, 2023^9^	Clinical trial	6 months	13-35 years	Toothpaste	T1: 6 months	T0	93	T0	52M/41F	probiotic	
						T1	85	T1	50M/35F	Novamin	
										Fluoride (control group)	

**Table 3:** A summary of the characteristics of the included in vitro studies

**Author / date**	**Study design**	**Novamin added to**	**Duration**	**Sample size**	**Groups**		**Time points**	
Abbassy 2019^10^	Invitro	paste		21 premolars	Novamin	Applied for 24 h		
					Fluoride	Applied for 5 min		
					Control	No treatment		
Al Shehab 2022^11^	Invitro	Paste (FBAG)		135 premolars (45 per group)	FBAG (BioMinF)			
					Alpha-Glaze (resin sealer)			
					Control (Transbond XT)			
Bakhsh 2017^12^	Invitro	Paste		45 premolars (15 per group)	BG	bioglass paste for 24 h		
					REM	remineralization solution for 24 h		
					CONT	no treatment		
Bakhsh 2018^13^	Invitro	Paste		15 premolars	REM	bioglass paste		
					CONTROL			
Bakry 2018^14^	Invitro	Paste		90 premolars	BioMinF (applied for 24 h)			
					Fluoride (4 min application)			
					Fluoride (24 h application)			
					Control (no treatment)			
Ballard 2013^15^	Invitro	Toothpaste	28 days	40 premolars	Control (artificial saliva)		pretreatment	
					Restore toothpaste (Novamin)		Immediately after demineralization	
					PreviDent 5000		Day 7 of treatment	
					MI paste plus		Day 14 of treatment	
							Day 21 of treatment	
							Day 28 of treatment	
Bichu 2013^16^	Invitro comparative	Paste		75 premolars	Control	No treatment		
					FP	Fluoride varnish		
					CPP-ACP			
					Novamin			
					CPP-ACP + fluoride			
Gokce 2017^17^	Invitro	Toothpaste	2 weeks	45 premolars	Fluoride-containing toothpaste (control)		T1 = Baseline: After 7 days of remineralization	
					Novamin-containing toothpaste		T2 = Immediately after 2 weeks of the treatment	
					Probiotic-containing toothpaste			
Mohanty 2014^18^	Invitro	toothpaste		40 premolars	Novamin (remineralizing paste)		T0	0 days
					Control		T1	2 days
							T2	10 days

###  Risk of bias assessment

 In the first part, the Cochrane Risk of Bias tool for randomized trials questionnaire (RoB 2) was used to investigate the risk of bias in clinical trials. The RoB 2 questionnaire has five domains, which include the following: risks of the randomization process, deviations from the intended interventions, missing outcome data, measurement of the outcome, and selection of the reported results. For each of the domains, according to the amount of information reported in the studies, a score of 2 (sufficient information), 1 (insufficient information), or 0 (no report) was assigned.

 Also, the quality and reliability of evidence and meta-analysis results were examined using the Grading of Recommendations Assessment, Development, and Evaluation ranking system (GRADE). The GRADE system evaluates the quality and reliability of evidence according to the type of articles (randomized, non-randomized), risk of bias, risk of non-uniformity of results, indirectness of evidence (the measured variable is not related to the objective), inaccuracy in the results (high probability of error in measuring the results), and other cases (printing bias, high difference between two groups, result dependent on intervention dose, presence of confounding variable). The quality and confidence of the evidence were classified into four categories: high, medium, low, and very low confidence (Table 4).

**Table 4:** GRADE assessment of certainly and quality of the evidence

**Certainty assessment**							**No of patients**		**Effect**		**Certainty**
**No. of studies**	**Study design**	**Risk of bias**	**Inconsistency**	**Indirectness**	**Imprecision**	**Other considerations**	**Novamin**	**[comparison]**	**Relative** **(95% CI)**	**Absolute** **(95% CI)**	
**Remineralization**											
3	Randomized trials	Serious	Not serious	not serious	not serious	none	73	73	-	SMD 0.29 SD lower (0.64 lower to 0.05 higher)	⨁⨁⨁◯ Moderate
**Lesion depth**											
3	In vitro studies	Serious	Serious	not serious	not serious	none	45	42	-	MD 63.98 micrometer lower (92.26 lower to 35.71 lower)	⨁◯◯◯ Very low

###  Statistical analysis 

 Due to the heterogeneity in the study and investigation method, random-effects inverse-variance meta-analysis was used to evaluate improvements caused by bioactive glass in WSLs. In the meta-analysis of clinical studies (the first part of the study), the amount of remineralization due to the use of bioactive glass was variable due to the continuous nature of the variable, and due to the difference in the range of evaluations, standard mean difference (SMD) was used. Also, in the review of in vitro studies (the second part of the study), mean difference (MD) was used to investigate the changes in the depth of WSLs due to the use of bioactive glass due to the continuous nature of the variable. Due to the lack of access to standard deviation (SD) in several laboratory studies, it was not possible to perform a meta-analysis on the ratio of calcium to phosphorus elements. Since the number of studies included in the meta-analysis was less than 10, it was not possible to use a funnel plot to check publication bias.

 Cochrane’s Q test was used to assess heterogeneity between studies, and the I^2^ test was used to measure the degree of non-continuity in pooled calculations due to heterogeneity between studies. I^2^ values < 30% indicate low heterogeneity, values between 30% and 60% mean heterogeneity, and values > 60% are considered significant heterogeneity. All analyses were performed using Review Manager 5.4 software. MD and 95% confidence interval were reported for all analyses. A *P* value of < 0.05 was considered significant for the analysis, but in the case of heterogeneity, the value of 0.1 was used due to low power.

## Results

###  Literature search results

 In this study, 718 articles were found through searches in databases MEDLINE: 449, Web of Science: 72, EMBASE: 96, Scopus: 84, Cochrane CENTRAL: 17, and six articles related to the study topic were collected through a hand search (724 articles in total). After removing 229 duplicate articles, the titles and abstracts of the remaining 495 articles (489 through databases and 6 through manual search) were reviewed.

 Of these, 460 articles were excluded due to the lack of coordination with the inclusion and exclusion criteria of our study: 23 animal studies, 19 studies with different interventions, 37 studies with different results, 13 studies with different study groups, 46 in vitro studies unrelated to the subject of the study; 52 review studies, and 270 studies unrelated to bioactive glass-containing toothpastes were excluded.

**Figure 1:**
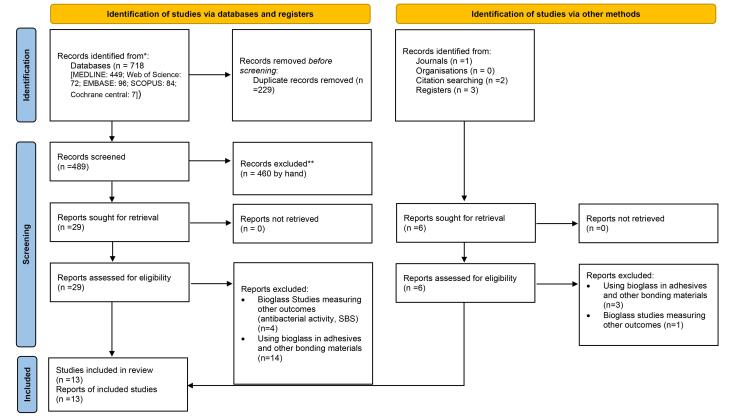
PRISMA study flow diagram

 The full texts of the remaining 35 studies (29 articles from databases and 6 articles by manual search) were retrieved and analyzed. Five studies were excluded due to the investigation of other properties of bioactive glasses (4 articles from databases and one article by manual search), and 17 studies were excluded due to the investigation of compounds containing bioactive glass except toothpaste and paste (such as bonding and adhesive) (14 articles from databases and 3 articles by manual search) (Figure 1).

 Among the remaining 13 articles, the study was divided into two parts.

###  The first part (clinical)

 Four articles that were conducted as clinical trials were included in our study for systematic review, and after data extraction, meta-analysis was performed on three articles, and for one study, meta-analysis was not performed due to the difference between the control group and other articles.

###  The second part (in vitro)

 Nine articles of studies that were conducted in vitro were included in our study for systematic review, and after data extraction, meta-analysis was performed on three articles, and meta-analysis was not performed for other articles due to the difference in the measurement index.

###  Characteristics of included studies

####  The first part

 Among all the clinical trial articles, three studies were selected for meta-analysis, which were conducted between 2015 and 2023. Table 2 presents the characteristics of the patients and the therapeutic interventions that have been performed for them.

 Unlike other studies, the study by Salah used CPP-ACP in the control group and was therefore excluded from the meta-analysis. Three other studies used fluoride toothpaste in the control group. The age range of the subjects was 12–35 years.

####  The second part

 In this section, the information from nine articles was reviewed, but due to the difference in the measurement index in these articles, only three articles could enter the meta-analysis.

 Three selected articles were completed between 2013 and 2022. The included studies evaluated the effectiveness of different toothpastes and pastes, including Novamin, fluoride, bioactive glass, CPP-ACP, and different toothpaste formulas. To simulate real dental conditions, these laboratory studies used 300 human tooth samples, i.e., premolars with orthodontic brackets.

 Overall, these studies evaluated the effect of treatments on remineralization, reduction of lesion depth, and improvements in the calcium/phosphate (Ca/P) ratio. The treatments were compared with different control groups, including untreated samples and fluoride and CPP-ACP in an artificial saliva medium. Table 3 presents the characteristics of the teeth and the therapeutic interventions that have been performed for them.

####  Risk of bias assessment

**Figure 2:**
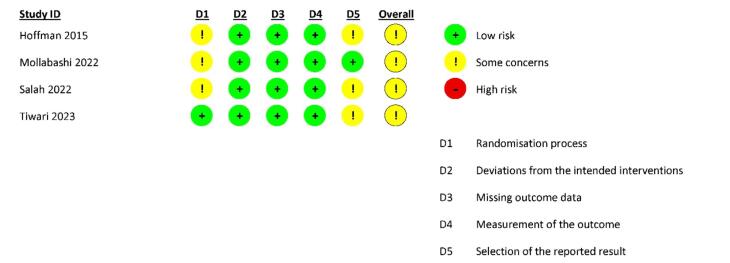
(a) Risk of bias summary of clinical studies (RoB 2); (b) risk of bias summary for in-vitro studies (QUIN)

 The results of the bias of the clinical studies reviewed with the ROB 2 tool are shown in Figure 2a. All clinical studies had some concerns about bias. Also, the bias of the in vitro studies evaluated with the QUIN tool is shown in Figure 2b. All the included studies had a moderate risk of bias. The input studies, especially regarding calculating the number of samples and presenting results, had a high risk of bias due to the lack of a predetermined protocol.

####  Meta-analysis 

 A random-effects meta-analysis was performed to investigate the effect of bioactive glass on white spots in both parts of the study (clinical and in vitro). In the first part, due to the difference in the method of measuring the remineralization of the studies, SMD was used to investigate the effect of BG on the white spot around the brackets. In the second part, the MD was used to check the depth of the lesion in laboratory studies. In these studies, dental samples with brackets were exposed to a demineralizing solution after toothpaste was used, and the lesion’s depth was investigated.

###  A meta-analysis of remineralization studies

####  The first part

**Figure 3:**
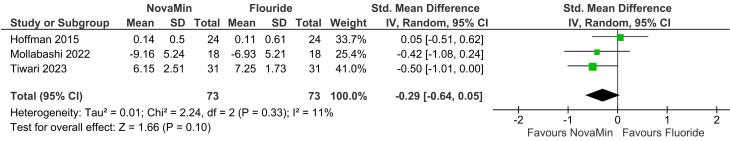
Forrest plot of random-effects analysis of differences between Novamin and fluoride

 In the meta-analysis of the remineralization of WSLs resulting from orthodontic treatment, 3 studies and 146 patients (73 patients treated with bioactive glass [Novamin] and 73 patients treated with fluoride) were studied, and no significant difference was observed between these two groups (*P* = 0.10, SMD = -0.29, 95% CI = -0.64, 0.05). During this analysis, low heterogeneity (l^2^ = 11%) was observed between the studies (Figure 3).

###  Meta-analysis examining the depth of the lesion

####  The second part

**Figure 4:**
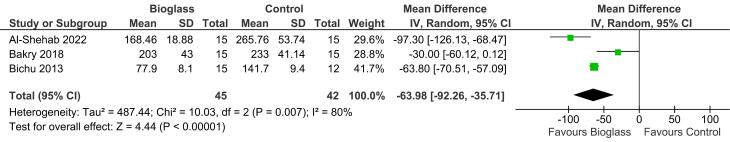
Forrest plot of random-effects analysis of differences between bioglass and control

 In the meta-analysis examining the depth of lesions before and after the impact of bioactive glass on extracted teeth, 3 studies and 87 teeth (45 teeth treated with bioactive glass and 42 teeth without treatment with bioactive glass or another substance) were examined. In the group treated with bioactive glass, the lesion depth after using a demineralization solution was less than in other groups, and this difference was statistically significant (*P* < 0.00001, MD = -63.98, 95% CI = -92.26, -35.71) (Figure 4).

 On average, the depth of lesions in this group was about 63 µm less after treatment. In performing this analysis, a high heterogeneity of 80% (I^2^ = 80%) was observed between the studies.

 The quality of evidence was evaluated using GRADE in the first part of clinical studies since the studies were RCTs. The low degree of heterogeneity of the study was attributed to the moderate risk of bias of the studies; in the second part of the in vitro studies, it was attributed to the high heterogeneity of the results. Moreover, the moderate risk of bias in the studies and the quality of studies was determined as “very low.” Table 4 presents the results of this study.

## Discussion

 This systematic review and meta-analysis examined the effect of bioactive glass-containing toothpastes on WSLs around orthodontic brackets, synthesizing evidence from clinical and in vitro studies. Thirteen studies met our inclusion, comprising four clinical trials and nine in vitro studies.

 In the first part of our analysis, clinical studies comparing the effects of bioactive glass-containing toothpastes to those of fluoride-containing toothpastes were evaluated. The meta-analysis found no statistically significant difference between the two groups, suggesting that bioactive glass-containing toothpaste can have a remineralizing effect on WSLs similar to fluoride toothpaste. This confirms the effectiveness of bioactive glass toothpastes but indicates no significant advantage over conventional fluoride toothpastes in clinical settings.

 In the second part of the systematic review, in vitro studies assessing the effect of bioactive glass-containing toothpastes on extracted teeth were analyzed. These studies included control groups that did not receive any treatment and experimental groups treated with bioactive glass. The results demonstrated that bioactive glass significantly reduced lesion depth compared to no treatment, supporting its potential benefit in managing WSLs.

 The variations in results between the clinical and in vitro studies can be attributed to factors like patient cooperation in the clinical part, which can influence study outcomes. In clinical studies, patients’ adherence to oral hygiene practices and the multifactorial nature of the oral cavity may impact the effectiveness of the treatment. In contrast, in vitro studies provide controlled environments that may not fully replicate clinical conditions.

 Bioactive glass has gained prominence in medicine and dentistry due to its ability to bond with bone and stimulate regeneration. Initially developed for bone regeneration, bioactive glasses have been incorporated into various dental products, including bonding agents,^19^ sealers,^20^ adhesives,^21^ and toothpaste. Our review focused on its application in toothpaste for treating WSLs.

 Clinical studies employed various methodologies to assess changes in WSLs resulting from treatment with bioactive glass-containing toothpaste. A common approach involved using the DIAGNOdent pen, which utilizes laser fluorescence to detect changes in tooth enamel. Fluorescence light is directed onto the WSLs before and after treatment, and the reflected light is measured, providing numerical values that indicate the extent of demineralization. These values allow for a quantitative analysis of the toothpaste’s effect on the lesions.

 Additionally, some studies calculated and compared the depth of lesions before and after treatment to directly measure the remineralization effect on subsurface enamel. For instance, Al-Shahab et al^11^ used transmitted light microscopy to calculate lesion depths and found a significant difference between the control and experimental groups. This method involves analyzing thin enamel sections under a microscope to assess demineralization precisely.

 Bakry et al^22^ and Hamba et al^23^ employed transverse microradiography (TMR), which is considered the gold standard for investigating the remineralization properties of compounds. In Bakry et al study, samples were sectioned into slices 100–120 µm in thickness, placed on x-ray-sensitive screens, and irradiated. The images were then digitized using a digital camera connected to a microscope for detailed calculations. However, they observed no significant difference between the control and experimental groups. In Hamba et al study, human molars with natural white-spot lesions were scanned for 5 min by µCT in different conditions: 50 µA, 165 µA, and 200 µA, with or without software beam-hardening correction (BHC). Thin sections at the same positions were then prepared for TMR. Lesion depth and mineral loss were compared between µCT and TMR. µCT measurements correlated well with TMR under all conditions, except for 0.5-mm Al without BHC. Similarly, Bichu et al^16^ used a trinocular research polarizing microscope to calculate lesion depth and reported a significant difference between the control and experimental groups.

 The varying results across clinical studies highlight the complexity of treating WSLs and the potential influence of multiple factors such as study design, sample size, and treatment duration. WSLs form in the subsurface layer of enamel, making them difficult to access for remineralization. Previous attempts to induce remineralization by optimizing calcium, phosphate, and fluoride concentrations have not yielded satisfactory results due to challenges in reaching deeper enamel layers.^24^

 In the in vitro studies, bioactive glass demonstrated promising results in promoting remineralization and inhibiting cariogenic bacteria. Compounds containing low or medium concentrations of fluoride are often insufficient to prevent or treat white spots, and high concentrations pose concerns regarding toxicity and adverse effects on the mechanical properties of nickel-titanium wires used in orthodontics.^25,26^ Moreover, high fluoride concentrations can inhibit the remineralization of subsurface enamel by forming a fluorapatite layer on the surface.^27^ These limitations underscore the importance of exploring alternative anti-caries compounds like bioactive glass.

 Bioactive glass can impact tooth decay through two primary mechanisms: an antibacterial effect on cariogenic bacteria and the remineralization of dental tissue.^4^ When bioactive glass dissolves in water, it releases alkaline ions that raise the pH, creating an environment hostile to *Streptococcus mutans*, a primary bacterium responsible for dental caries.^28^ The dissolution process leads to the controlled release of calcium, phosphate, and fluoride ions, which bind to the tooth surface, forming and crystallizing into fluoroapatite.^29^ The structure of silicate glasses can be designed to control the rate of dissolution, which accelerates under acidic conditions due to increased hydrogen ion concentration.

 The release of calcium and phosphate from bioactive glass upon contact with water results in a rise in pH, causing these ions to form a layer on the lesion surface along with ions in saliva.^30^ This new layer exhibits good wear resistance and eventually transforms into hydroxyapatite, structurally similar to natural enamel and dentin.^31^ In addition to remineralization capabilities, bioactive glass positively affects gingival health, as noted by Tai et al.^32^ The calcium and sodium content influences the bacterial balance in the oral environment, contributing to overall oral health.^33^

 Several studies support the potential of bioactive glass in promoting remineralization. Dai et al^4^ conducted a systematic review of 23 articles, finding that bioactive glass can prevent cariogenic bacteria growth and induce remineralization by forming apatite on demineralized enamel and dentin surfaces. Similarly, Alamri et al^29^ reviewed seven in vitro studies, concluding that resin-based dental materials with bioactive glass had significantly greater anti-demineralization properties than those without.

 However, other studies suggest no significant advantage of bioactive glass over conventional treatments. Khijmatgar et al^34^ found no significant difference between toothpastes with or without Novamin, recommending further studies. In a clinical trial, Hoffman et al^6^ also reported no significant differences in WSLs, plaque levels, or gingival health between bioactive glass and fluoride-containing toothpaste.

## Strengths and Limitations

 One of the strengths of this study is the comprehensive electronic and manual search of studies. Also, the quality of evidence was evaluated using GRADE. Another strength of this study is the simultaneous review of clinical and in vitro studies.

 One of the weaknesses of our study is the high heterogeneity of meta-analysis of in vitro studies. Also, the number of studies included in the meta-analysis was small, so it is suggested that other compounds containing bioactive glass, such as adhesives and bonding agents, should be investigated to achieve more comprehensive results in future studies. It is also recommended that these toothpastes be administered to patients during orthodontic treatment, and the white spots should be investigated in a prospective study due to the lack of reported side effects.

## Conclusion

 Bioactive glass-containing toothpastes can cause remineralization of WSLs around orthodontic brackets and improve oral hygiene. However, the results of these toothpastes did not show significant differences from toothpastes containing fluoride.

## Competing Interests

 None to declare.

## Ethical Approval

 The study was approved by the ethics committee of MUMS (Ethical approval number: IR.MUMS.DENTISTRY.REC.1401.063).

## References

[1] Selwitz RH, Ismail AI, Pitts NB (2007). Dental caries. Lancet.

[2] Temel SS, Kaya B (2019). Diagnosis, prevention and treatment of white spot lesions related to orthodontics. Int J Oral Dent Health.

[3] Taha AA, Patel MP, Hill RG, Fleming PS (2017). The effect of bioactive glasses on enamel remineralization: a systematic review. J Dent.

[4] Dai LL, Mei ML, Chu CH, Lo EC (2019). Mechanisms of bioactive glass on caries management: a review. Materials (Basel).

[5] Milly H, Festy F, Watson TF, Thompson I, Banerjee A (2014). Enamel white spot lesions can remineralise using bio-active glass and polyacrylic acid-modified bio-active glass powders. J Dent.

[6] Hoffman DA, Clark AE, Rody WJ Jr, McGorray SP, Wheeler TT (2015). A prospective randomized clinical trial into the capacity of a toothpaste containing NovaMin to prevent white spot lesions and gingivitis during orthodontic treatment. Prog Orthod.

[7] Mollabashi V, Heydarpour M, Farhadifard H, Alafchi B (2022). DIAGNOdent pen quantification of the synergy of NovaMin® in fluoride toothpaste to remineralize white spot lesions in patients with fixed orthodontic appliances: a double-blind, randomized, controlled clinical trial. Int Orthod.

[8] Salah R, Afifi RR, Kehela HA, Aly NM, Rashwan M, Hill RG (2022). Efficacy of novel bioactive glass in the treatment of enamel white spot lesions: a randomized controlled trial. J Evid Based Dent Pract.

[9] Tiwari A, Jain RK (2023). Comparative evaluation of white spot lesion incidence between NovaMin, probiotic, and fluoride containing dentifrices during orthodontic treatment using laser fluorescence - a prospective randomized controlled clinical trial. Clin Investig Orthod.

[10] Abbassy MA, Bakry AS, Alshehri NI, Alghamdi TM, Rafiq SA, Aljeddawi DH (2019). 45S5 Bioglass paste is capable of protecting the enamel surrounding orthodontic brackets against erosive challenge. J Orthod Sci.

[11] Al Shehab A, Bakry AS, Hill R, Alsulaimani FF, Abbassy MA (2022). Evaluation of bioactive glass and low viscosity resin as orthodontic enamel sealer: an in vitro study. J Funct Biomater.

[12] Bakhsh TA, Bakry AS, Mandurah MM, Abbassy MA (2017). Novel evaluation and treatment techniques for white spot lesions An in vitro study. Orthod Craniofac Res.

[13] Bakhsh T, Al-Batati M, Mukhtar M, Al-Najjar M, Bakhsh S, Bakhsh A, et al. Effect of bioglass on artificially induced enamel lesion around orthodontic brackets: OCT study. In: Lasers in Dentistry XXIV. San Francisco, CA: SPIE; 2018. p. 9-17. 10.1117/12.2285936.

[14] Bakry AS, Abbassy MA, Alharkan HF, Basuhail S, Al-Ghamdi K, Hill R (2018). A novel fluoride containing bioactive glass paste is capable of re-mineralizing early caries lesions. Materials (Basel).

[15] Ballard RW, Hagan JL, Phaup AN, Sarkar N, Townsend JA, Armbruster PC (2013). Evaluation of 3 commercially available materials for resolution of white spot lesions. Am J Orthod Dentofacial Orthop.

[16] Bichu YM, Kamat N, Chandra PK, Kapoor A, Razmus T, Aravind NK (2013). Prevention of enamel demineralization during orthodontic treatment: an in vitro comparative study. Orthodontics (Chic).

[17] Gokce G, Savas S, Kucukyilmaz E, Veli I (2017). Effects of toothpastes on white spot lesions around orthodontic brackets using quantitative light-induced fluorescence (QLF): an in vitro study. J Orofac Orthop.

[18] Mohanty P, Padmanabhan S, Chitharanjan AB (2014). An in vitro evaluation of remineralization potential of NovaMin® on artificial enamel sub-surface lesions around orthodontic brackets using energy dispersive X-ray analysis (EDX). J Clin Diagn Res.

[19] Manfred L, Covell DA, Crowe JJ, Tufekci E, Mitchell JC (2013). A novel biomimetic orthodontic bonding agent helps prevent white spot lesions adjacent to brackets. Angle Orthod.

[20] Abbassy MA, Bakry AS, Hill R (2021). The efficiency of fluoride bioactive glasses in protecting enamel surrounding orthodontic bracket. Biomed Res Int.

[21] Chaichana W, Insee K, Chanachai S, Benjakul S, Aupaphong V, Naruphontjirakul P (2022). Physical/mechanical and antibacterial properties of orthodontic adhesives containing Sr-bioactive glass nanoparticles, calcium phosphate, and andrographolide. Sci Rep.

[22] Bakry AS, Abbassy MA (2018). Increasing the efficiency of CPP-ACP to remineralize enamel white spot lesions. J Dent.

[23] Hamba H, Nikaido T, Sadr A, Nakashima S, Tagami J (2012). Enamel lesion parameter correlations between polychromatic micro-CT and TMR. J Dent Res.

[24] Hamba H, Nikaido T, Inoue G, Sadr A, Tagami J (2011). Effects of CPP-ACP with sodium fluoride on inhibition of bovine enamel demineralization: a quantitative assessment using micro-computed tomography. J Dent.

[25] Abbassy MA (2016). Fluoride influences nickel-titanium orthodontic wires’ surface texture and friction resistance. J Orthod Sci.

[26] Gupta AK, Shukla G, Sharma P, Gupta AK, Kumar A, Gupta D (2018). Evaluation of the effects of fluoride prophylactic agents on mechanical properties of nickel-titanium wires using scanning electron microscope. J Contemp Dent Pract.

[27] Bergstrand F, Twetman S (2011). A review on prevention and treatment of post-orthodontic white spot lesions - evidence-based methods and emerging technologies. Open Dent J.

[28] Xu YT, Wu Q, Chen YM, Smales RJ, Shi SY, Wang MT (2015). Antimicrobial effects of a bioactive glass combined with fluoride or triclosan on Streptococcus mutans biofilm. Arch Oral Biol.

[29] Alamri A, Salloot Z, Alshaia A, Ibrahim MS (2020). The effect of bioactive glass-enhanced orthodontic bonding resins on prevention of demineralization: a systematic review. Molecules.

[30] Rajan R, Krishnan R, Bhaskaran B, Kumar SV (2015). A polarized light microscopic study to comparatively evaluate four remineralizing agents on enamel viz CPP-ACPF, ReminPro, SHY-NM and Colgate strong teeth. Int J Clin Pediatr Dent.

[31] Sauro S, Thompson I, Watson TF (2011). Effects of common dental materials used in preventive or operative dentistry on dentin permeability and remineralization. Oper Dent.

[32] Tai BJ, Bian Z, Jiang H, Greenspan DC, Zhong J, Clark AE (2006). Anti-gingivitis effect of a dentifrice containing bioactive glass (NovaMin®) particulate. J Clin Periodontol.

[33] Shihabi S, AlNesser S, Comisi JC (2021). Comparative remineralization efficacy of topical NovaMin and fluoride on incipient enamel lesions in primary teeth: scanning electron microscope and Vickers microhardness evaluation. Eur J Dent.

[34] Khijmatgar S, Reddy U, John S, Badavannavar AN, T DS (2020). Is there evidence for NovaMin application in remineralization?: A systematic review. J Oral Biol Craniofac Res.

